# Treatment Strategy for Petroclival Meningiomas Based on a Proposed Classification in a Study of 168 Cases

**DOI:** 10.1038/s41598-020-61497-y

**Published:** 2020-03-13

**Authors:** Zijin Zhao, Xianrui Yuan, Jian Yuan, Li Cai, Weixi Jiang, Yuanyang Xie, Siyi Wanggou, Chi Zhang, Guodong Tang, Haoyu Li, Zefeng Peng, Xuejun Li, Qing Liu

**Affiliations:** 1 Department of Neurosurgery, Xiangya Hospital, Central South University, 87 Xiangya Road, Changsha, Hunan 410008 P R China; 2 Neurosurgical Institute, Central South University, 87 Xiangya Road, Changsha, Hunan 410008 P R China; 3 The Institute of Skull Base Surgery and Neurooncology at Hunan, 87 Xiangya Road, Changsha, Hunan 410008 P R China; 4 Department of Neurosurgery, The First Affiliated Hospital of University of South China, 69 Chuanshan Road, Hengyang, Hunan 421000 P R China; 5 Arkansas Neuroscience Institute, St. Vincent Hospital, 6101 Saint Vincent Cir, Little Rock, Arkansas, AR 72205 United States

**Keywords:** CNS cancer, Neurology

## Abstract

Petroclival meningiomas (PCMs) are regarded as one of the most formidable challenges in neurosurgery. We retrospectively assessed the surgical outcomes of PCMs based on a tumor classification to evaluate the long-term outcomes. A series of 168 patients with PCMs from July 1996 to January 2017. On the basis of the difference in the origin of dural attachment and patterns of growth, the PCMs were classified into 4 different types. The clinical characteristics, surgical record, and follow-up data of each type were reviewed. The study included 138 females (82.1%) with an average age of 49.9 ± 16.2 years. And 138 cases (82.1%) had developed neurological deficits preoperatively with the average tumor size of 44.0 ± 10.6 mm. Specific surgical approaches were applied depended on the tumor classification. Gross total resection (GTR) was achieved in 119 cases (70.8%) with the complications of 46 cases (27.7%). With a median follow-up of 86.5 months, there were 41 cases of recurrence/progress (25.7%) and 39 cases of morbidity (26.4%). Compared with the non-GTR group, the GTR significantly decreased the R/P rate (P = 0.001), prolonged the R/P-FS time (P = 0.032) and improved the follow-up neurological status (P = 0.026). Favorable outcomes and acceptable morbidity were achieved with the treatment strategy of the choice of specific approaches for each type. Meanwhile, the differences of each type in diverse clinical characteristic were verified. Individualized assessment and suitable approach choice should be based on the tumor classification to improved the GTR and quality of life for patients.

## Introduction

Petroclival meningiomas (PCMs) arise from the upper two-thirds of the clivus, are located at the petroclival junction, medial to the internal auditory meatus (IAM), and posterior to the trigeminal nerve. PCMs are regarded as one of the most formidable challenges in neurosurgery because of their proximity to the brainstem and vital and eloquent neurovascular structures, which definitely determine the difficulty to perform gross total resection (GTR) and lead to high rates of surgical morbidity and mortality. More attention has been paid to the relationship between tumor classification and the choice of surgical approach to find more favorable outcomes, and various tumor classifications in the petroclival region have been recommended^1–6^. On the basis of the different origins of the dural attachment of the lesion, growth patterns, and the circumjacent range involved, we classified PCMs into four different anatomical types which was regarded as matching the variation of the pathologic anatomy in the petroclival region. The purpose of this study is to evaluate the efficacy and suitability of the surgical approach choice based on the tumor classification and our experience of over 20 years in managing PCM patients, to better understand the role of various surgical approaches in order to explore a more ideal individualized treatment strategy for PCMs.

## Patients and methods

### Patient population

In this retrospective study, 168 cases of WHO grade I PCMs were treated with microsurgery from July 1996 to January 2017 by the same senior surgeon. The clinical charts, neuroimaging, operation records, and follow-up data were reviewed. The neurological assessments included cranial nerve (CN) deficits, ataxia, and hemiparesis. All the principal signs and symptoms presented were obtained from the medical records. The Karnofsky Performance Scale (KPS) score was taken for the neurological function status on admission, two weeks postoperatively, and at follow-up visits. The Research Ethics Committee of the Xiangya Hospital of Central South University approved this study.

### Tumor classification

**Figure 1:**
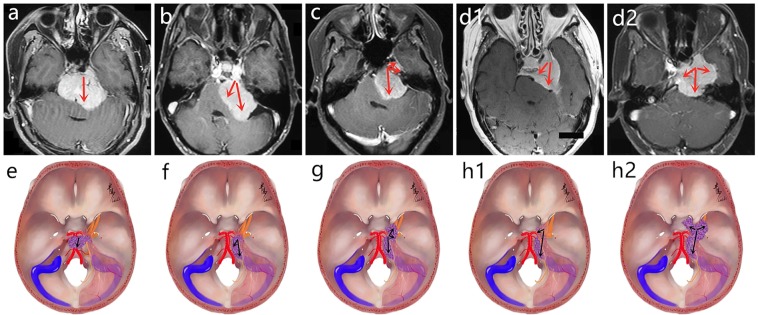
The proposed classification of PCMs. (**a**,**e**) **Clivus type:** The dural attachment originates from the intradural part of the petroclival fissure, and the main part of the lesion is located in the middle-upper clivus, mainly grows toward the middle line or even the contralateral region. (**b**,**f**) **Petroclival type:** The dural attachment also originates from petroclival fissure, but mainly grows toward the homolateral dorsal petrosum region, and the main part is located in the middle clivus and expands toward the cerebellopontine angle region. (**c**,**g**) **Petroclivosphenoidal type:** The growth pattern is mainly from the posterior cranial fossa to middle cranial fossa; the dural attachment originates from the middle-upper clivus, expands forward and upward along the petroclival fissure, and extends to the dorsum sellae and parasellar region or arrives at the posterior wall of CS. (**d1,h1**) **Sphenopetroclival subtype I:** The lesion totally originates within the CS without encroaching the sinus wall resulting in the sinus area expansile hyperplasia; the lateral wall of the lesion is smooth maintaining the dural space between the lesion and temporal lobe. **(d2,h2) Sphenopetroclival subtype II:** The lesion originates on the lateral wall of CS and grows toward both outside and inside of sinus, and partial lesion could reach the sphenoclival fissure, expand into the parasellar region, and invade into the PCF; the sinus wall is rough and dural space is unclear or even disappeared between the lesion and temporal lobe. The red arrows and black arrows showed the growth pattern of lesions in different types.

On the basis of the changes in the pathologic anatomy following: (1) the different origin of dural attachment of lesions; (2) the different possible growth patterns; (3) the involvement of circumjacent structures such as Meckel’s cave (MC), cavernous sinus (CS), and IAM from radiography data and intraoperative findings, we classified the PCMs into 4 different types and 2 subtypes as follows (Fig. 1).

**Figure 2:**
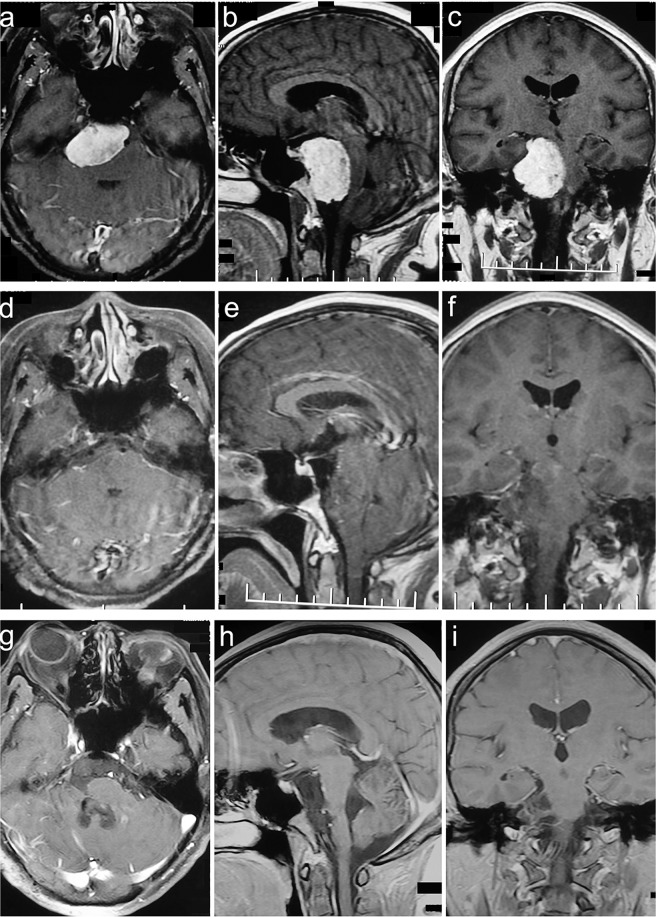
Clivus type case. A 56-year-old female presented with headache, dysphagia, bucking and hearing impairment for 84 months. The preoperative KPS score was 80. She was achieved GTR with the RSA of palsy in CN V and VII. The postoperative KPS score was 50. With a follow-up of 75 months, she participated in normal activities and had a KPS score of 90 without recurrence. (**a–c**) Preoperative MRI T1 contrast axial, sagittal, and coronal images. (**d–f**) Postoperative MRI T1 contrast axial, sagittal, and coronal images. (**g–i**) Follow-up MRI T1 contrast axial, sagittal, and coronal images.

**Figure 3:**
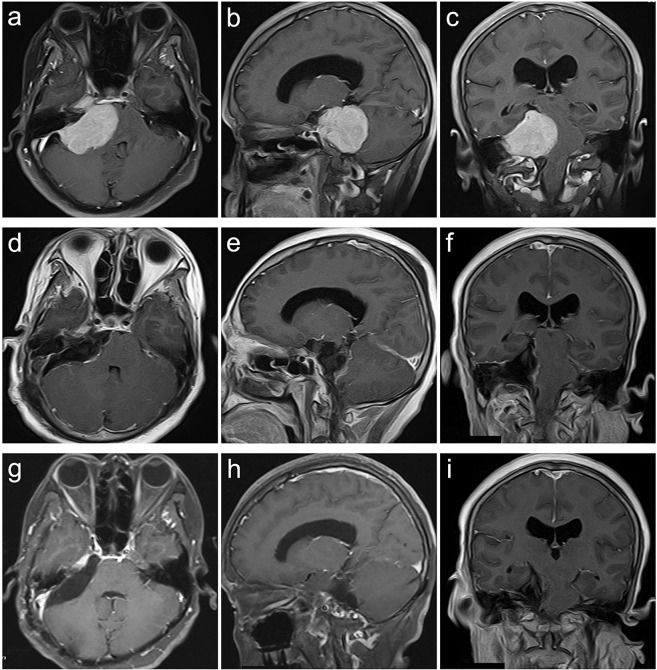
Petroclival type case. A 47-year-old female presented with headache, dysphagia, bucking, hearing impairment and ataxia for 36 months. The preoperative KPS score was 60. She was achieved GTR with the RTTA of palsy in CN V VI,VI and VII and gastric intubation. The postoperative KPS score was 50. With a follow-up of 24 months, she participated in normal activities and had a KPS score of 80 without recurrence. (**a–c**) Preoperative MRI T1 contrast axial, sagittal, and coronal images. (**d–f**) Postoperative MRI T1 contrast axial, sagittal, and coronal images. (**g–i**) Follow-up MRI T1 contrast axial, sagittal, and coronal images.

**Figure 4:**
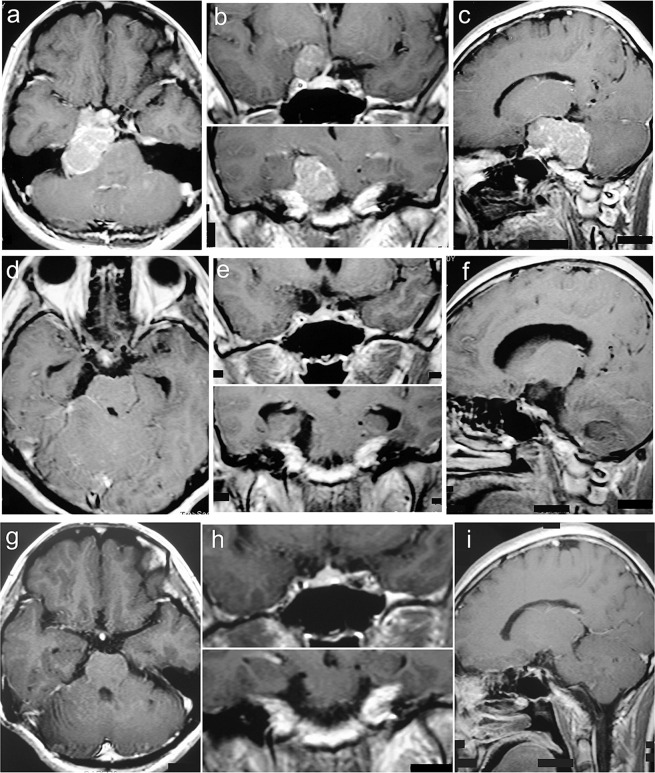
Petroclivosphenoidal type case 1. A 40-year-old female presented with headache and prosopalgia for 60 months. The preoperative KPS score was 70. She was achieved GTR with the RISA of palsy in CN V. The postoperative KPS score was 80. With a follow-up of 108 months, she participated in normal activities and had a KPS score of 90 without recurrence. (**a–c**) Preoperative MRI T1 contrast axial, sagittal, and coronal images. (**d–f**) Postoperative MRI T1 contrast axial, sagittal, and coronal images. (**g–i**) Follow-up MRI T1 contrast axial, sagittal, and coronal images.

**Figure 5:**
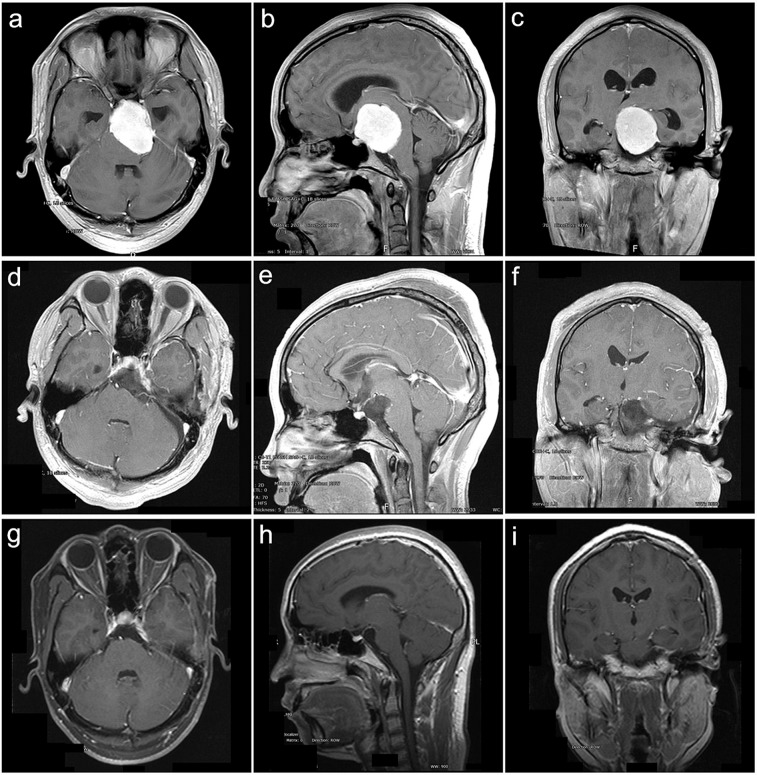
Petroclivosphenoidal type case 2. A 58-year-old female presented with headache and hobble for 8 months. The preoperative KPS score was 80. She was achieved GTR with the STTA of palsy in CN III, IV and V. The postoperative KPS score was 70. With a follow-up of 48 months, she participated in normal activities and had a KPS score of 90 without recurrence. (**a–c**) Preoperative MRI T1 contrast axial, sagittal, and coronal images. (**d–f**) Postoperative MRI T1 contrast axial, sagittal, and coronal images. (**g–i**) Follow-up MRI T1 contrast axial, sagittal, and coronal images.

**Figure 6:**
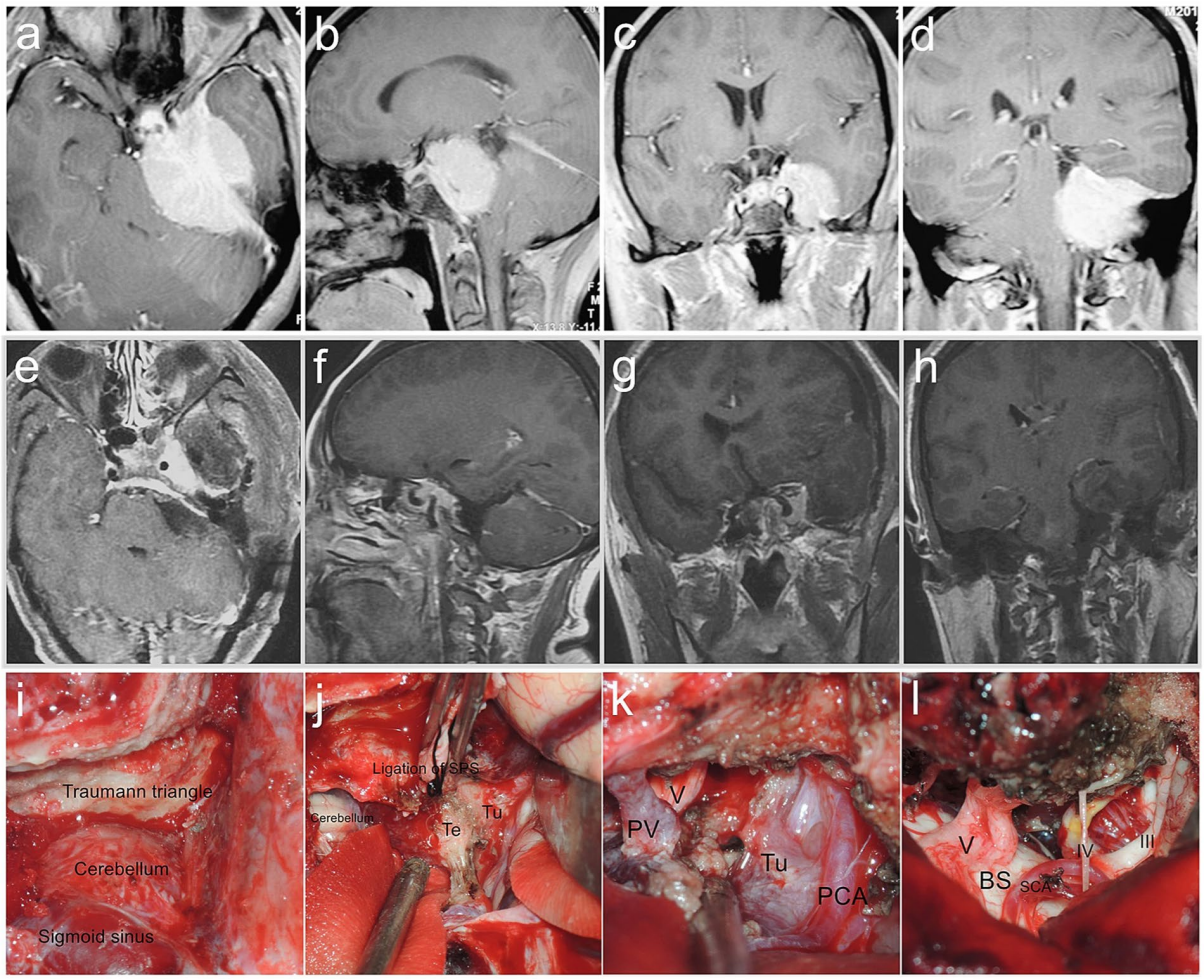
Petroclivosphenoidal type case 3. A 44-year-old male presented with headache prosopalgia and hobble for 18 months. The preoperative KPS score was 70. He was achieved STR with the PCA of palsy in CN V, VII and VIII. The postoperative KPS score was 50. And he received the GKS after discharge of 1 month. (**a–d**) Preoperative MRI T1 contrast axial, sagittal, and coronal images. (**e–h**) Postoperative MRI T1 contrast axial, sagittal, and coronal images. (**i–l**) Intraoperative figures demonstrating steps of petroclivosphenoidal type resection via the PCA. (**i**) Exposure of the traumann triangle, cerebellum, sigmoid sinus and transverse sinus. (**j**) Ligation of the superior petrosal sinus and incision of the tentorium to expose the supra-infratentorial structures and tumor. (**k**) Tumor resection between the multiple intervals of the neurovascular structures. (**l**) Subtotal tumor resection and keeping well the brain stem and neurovascular structures integrity. SPS, Superior Petrosal Sinus; SCA, Superior Cerebellar Artery; PCA, Posterior Cerebral Artery; Tu, Tumor; PV, Petrosal Vein; Te, Tentorium; BS, Brainstem.

**Figure 7:**
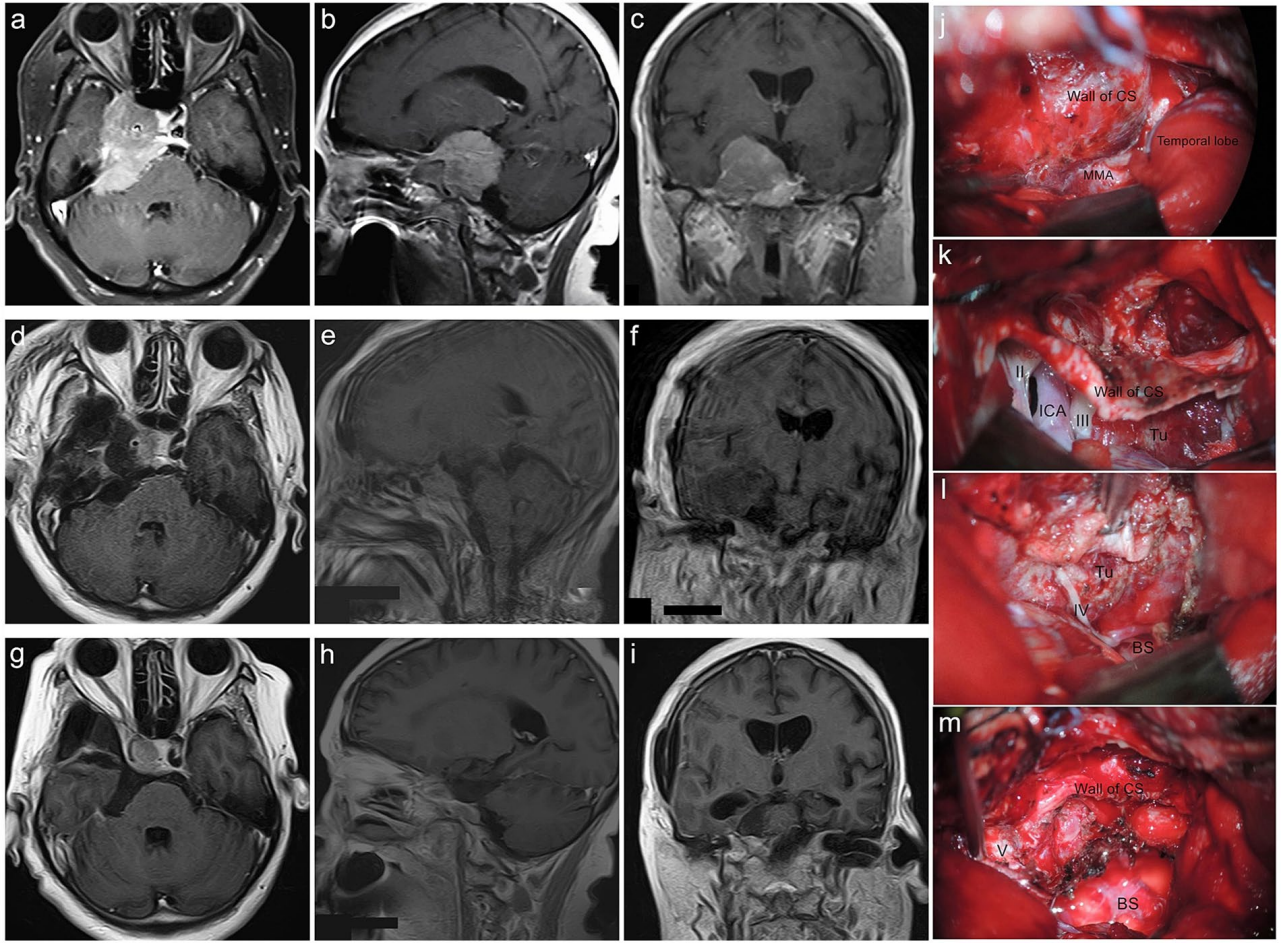
Sphenopetroclival subtype I case. A 40-year-old female presented with facial numbness, altered ocular motility and hearing impairment for 48 months. The preoperative KPS score was 90. She was achieved GTR with the PTCA of palsy in CN III and VI and intracranial infection. The postoperative KPS score was 50. With a follow-up of 45 months, she participated in normal activities and had a KPS score of 90 without recurrence. (**a–c**) Preoperative MRI T1 contrast axial, sagittal, and coronal images. (**d–f**) Postoperative MRI T1 contrast axial, sagittal, and coronal images. (**g–i**) Follow-up MRI T1 contrast axial, sagittal, and coronal images. (**j–m**) Intraoperative figures demonstrating steps of sphenopetroclival subtype I resection via the PTCA. (**j**) Exposure of the wall of the cavernous sinus within part of tumor and middle meningeal artery after temporal lobe retraction. (**k**) Coagulation of the middle meningeal artery, incision of wall of cavernous sinus to remove the tumor within the sinus, then incision of the dura mater to expose the subdural structures and tumor. (**l)** Subdural tumor resection between the multiple intervals of the neurovascular structures. (**m**) Total tumor resection and keeping well the brain stem and neurovascular structures integrity. MMA, Middle Meningeal Artery; ICA, Internal Carotid Artery; Tu, Tumor; BS, Brainstem.

**Figure 8:**
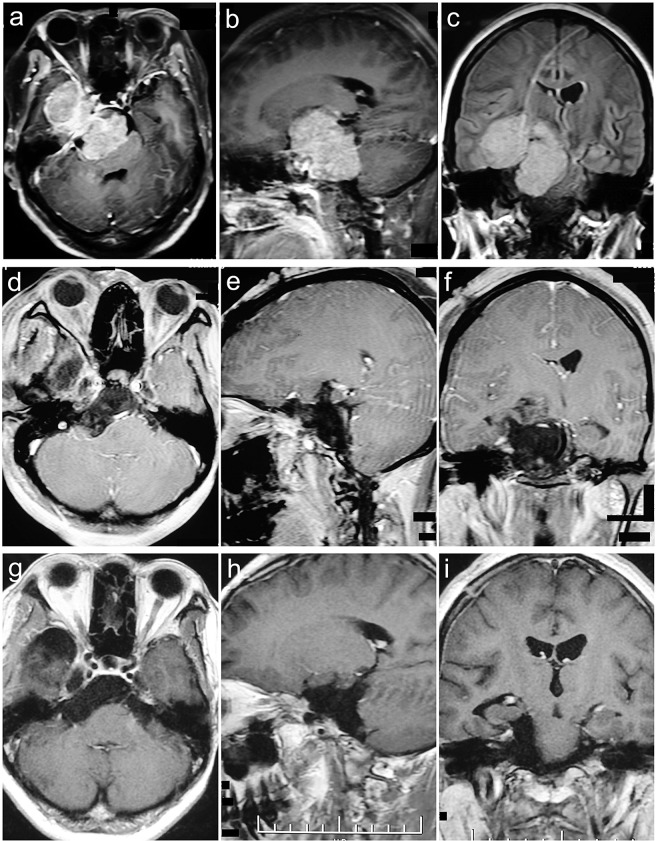
Sphenopetroclival subtype II case. A 41-year-old female presented with epilepsy for 7 months. The preoperative KPS score was 70. She was achieved GTR with the EPTA. The postoperative KPS score was 60. With a follow-up of 30 months, she participated in normal activities and had a KPS score of 90 without recurrence. **(a–c**) Preoperative MRI T1 contrast axial, sagittal, and coronal images. (**d–f**) Postoperative MRI T1 contrast axial, sagittal, and coronal images. (**g–i**) Follow-up MRI T1 contrast axial, sagittal, and coronal images.

- **Clivus type** (CV type): The dural attachment originates from the intradural part of the petroclival fissure (PF), and the main part of the lesion locates in the middle-upper clivus, mainly grows toward the middle line or even the contralateral region, and compresses the brainstem backward (Fig. 2).
- **Petroclival type** (PC type): The dural attachment also originates from the PF, but mainly grows toward the homolateral dorsal petrosum region, and the main part locates in the middle clivus and expands toward the cerebellopontine angle region (Fig. 3).
- **Petroclivosphenoidal type** (PC-S type): The growth pattern is mainly from the posterior cranial fossa (PCF) to the middle cranial fossa (MCF) and from the infratentorial to supratentorial compartment. The dural attachment originates from the middle-upper clivus, expands forward and upward along the PF, and extends to the dorsum sellae, posterior clinoid process, and parasellar region by crossing the petrous ridge, or invades into MC and even arrives at the posterior wall of CS by crossing through MC (Figs. 4–6).
- **Sphenopetroclival type** (S-PC type): The growth pattern is mainly from the MCF to PCF with the site of origin saddling the petrous ridge and invading the CS broadly. Then, depending on the relationship between the origin of dural attachment and CS, the type is further divided into two subtypes.
- **Subtype I** (S-PC I type): The lesion totally originates within the CS without encroachng the sinus wall leading to the CS area expansile hyperplasia, and only part of the lesion expanding into the PCF through the petrous apex. The lateral wall of the lesion is smooth and maintains the dural space between the lesion and temporal lobe (Fig. 7).
- **Subtype II** (S-PC II type): The lesion originates on the lateral wall of CS and grows both outside and inside of sinus, resulting in the sinus wall is rough and dural space is unclear or even disappeared between the lesion and temporal lobe; partial lesions could reach the sphenoclival fissure, expand into the parasellar region, dorsum sellae, and petrous apex, and invade into the PCF (Fig. 8).

### Surgical management and follow-up

The surgical approach selection was mainly based on tumor classification. Meanwhile, patient factors such as age, appeals and preoperative condition were also considered. Intraoperative neurophysiological monitoring including motor evoked potentials, somatosensory evoked potentials, brainstem auditory evoked potentials and cranial nerve electromyography were routinely performed. The extent of tumor removal (EOR) was classified into three degrees, depending on the postoperative contrast MRI and intraoperative findings including GTR (Simpson Grades I/II), subtotal resection (STR) (Simpson Grades III/IV, with 90–99% excision of the lesion) and partial resection (PR) (Simpson III/IV, with below 90% excision of the lesion). Preoperative, postoperative and follow-up quality of life (QOL) were all measured by using KPS score evaluated by two neurosurgeons independently. The excellent QOL was defined as the KPS score ≥80 and the patient could work and live independently and normally. The recurrence/progress (R/P) meant tumor regrowth *in situ* after GTR and residual tumor regrowth after STR/PR if the increase of the maximal diameter was larger than 3 mm. The follow-up investigation was performed at 3 and 6 months after surgery and then once each 1 or 2 years via clinic visits in most cases. And the questionnaires were also used for some patients. The latest follow-up deadline was May 1st, 2018.

### Statistical analysis

A descriptive analysis of data was performed using the IBM SPSS Statistical Package 21.0. Probability value < 0.05 indicated statistical significance. A univariate analysis was used to evaluate the clinical data (paired samples t test or Chi-square test) and the R/P of lesion (Kaplan-Meier survival analysis).

### Compliance with ethical standards

All procedures performed in studies involving human participants were in accordance with the ethical standards of the institutional and/or national research committee and with the 1964 Helsinki Declaration and its later amendments or comparable ethical standards. And informed consent was obtained from all individual participants included in the study.

### Informed consent

Informed consent was obtained from all individual participants included in the study.

## Results

### Patient features

**Figure 9:**
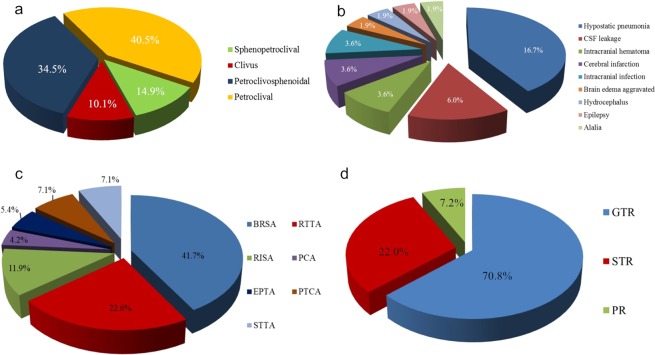
The Pie chart showed the constituent ratio of the different clinical data and outcomes. (**a**) The constituent ratio of different tumor types proposed. (**b**) The constituent ratio of the postoperative complications. (**c**) The constituent ratio of the different choice of surgical approaches. (**d**) The constituent ratio of the extent of tumor removal.

A total of 168 patients were enrolled including 30 males (17.9%) and 138 females (82.1%) with an average and median age of 49.9 ± 16.2 and 51.0 years (range 15–73 years), respectively. A total of 152 cases (90.5%) underwent initial microsurgery, with 12 cases of prior surgeries and 4 cases of prior gamma knife radiosurgery (GKS). The initial clinical manifestations were versatile including headache and dizziness (58 cases, 34.5%), facial numbness (34 cases, 20.2%), hearing loss (22 cases, 13.1%), prosopalgia (24 cases, 14.3%), unstable walking (16 cases, 9.5%), and tinnitus (14 cases, 8.3%) with the mean and median preoperative symptom duration of 37.6 ± 18.3 and 41.2 months (range 1 week to 360 months), respectively. Preoperatively, 138 cases (82.1%) had developed neurological deficits (Table 1). Based on the tumor classification, the PCMs included the CV type of 17 cases (10.1%), PC type of 68 cases (40.5%), PC-S type of 58 cases (34.5%), S-PC I type of 14 cases (8.3%) and S-PC II type of 11 cases (6.8%) (Fig. 9a). All of clinical data was stratified based on tumor type (Table 2). The mean of maximum tumor diameter was 44.0 ± 10.6 mm (range 15–80 mm) with 96.4% of large (25–44 mm, 78 cases) and giant (≥45 mm, 84 cases) according to the Sekhar standard^7^. The preoperative KPS score was on average 73.7 ± 13.6 (range 40–100). The lesions of S-PC type were significantly larger than the other types (P = 0.004) and showed more CS involvement (P = 0.013); the PC-S type showed the highest rate of brainstem edema (P = 0.039) (Table 2).

**Table 1:** Neurological deficits analysis.

Deficits	Preoperation (n = 168)	Postoperation (n = 166)		Follow-Up (n = 148)	
	Neurological Deficit	Surgical morbidity	Improvement	No Change	Improvement or Recovery
**Total (%)**	**138 (82.1)**	**68 (40.5)**	**62 (37.3)**	**39 (26.4)**	**84 (56.8)**
Ataxia	61	24	36	13	52
Hemiparesis	19	14	7	22	11
CNIII	14	32	4	17	15
CNIV	26	38	—	32	6
CNV	84	11	23	28	30
CNVI	14	26	3	20	6
CNVII	20	34	5	12	22
CNVIII	38	20	13	25	16
CNIX-XII	58	6	22	32	36

**Table 2:** Clinical characteristics of PCMs categorized based on tumor type*.

Variable	CV	PC	PC-S	S-PC	Total	*t / χ*^2^	p Value
Patients No. (%)	17 (10.1)	68 (40.5)	58 (34.5)	25 (14.9)	168	—	—
Female sex (%)	14 (82.4)	55 (80.9)	49 (84.5)	20 (80.0)	138 (82.1)	0.895	0.784
Patient age, yrs	46.2 ± 10.5	49.5 ± 9.8	50.0 ± 10.5	53.7 ± 9.1	49.9 ± 10.5	1.543	0.365
Tumor size, mm	43.0 ± 10.4	42.6 ± 10.3	44.7 ± 10.6	47.35 ± 10.4	44.0 ± 10.6	**8.456**	**0.004**^**†**^
CS invasion (%)	3 (17.6)	23 (33.8)	19 (32.8)	14 (56.0)	59 (35.1)	**6.178**	**0.013**^**†**^
NVS** encasement (%)	7 (41.2)	30 (44.1)	27 (46.6)	14 (56.0)	78 (46.4)	0.889	0.546
Brainstem edema (%)	3 (17.6)	15 (22.1)	24 (41.4)	6 (24.0)	48 (28.6)	**4.282**	**0.039**^**†**^
Hydrocephalus (%)	6 (35.3)	26 (38.2)	23 (39.7)	9 (36.0)	64 (38.1)	0.039	0.844
GTR (%)	13 (76.5)	51 (75.0)	39 (67.2)	16 (64.0)	119 (70.8)	**10.126**	**0.001**^**†**^
STR (%)	3 (17.6)	13 (19.1)	14 (24.1)	7 (28.0)	37 (22.0)	**9.813**	**0.002**^**†**^
Surgical duration, hrs	5.8 ± 1.2	6.2 ± 1.7	6.5 ± 2.1	7.2 ± 2.2	6.4 ± 1.8	**4.292**	**0.006**^**†**^
Postoperative LOS, dys	13.6 ± 8.3	14.4 ± 8.9	15.3 ± 8.0	17.2 ± 9.0	15.1 ± 8.6	**2.873**	**0.017**^**†**^
Postoperative complications (%) (n = 166)	4 (23.5)	18 (26.9)	16 (28.1)	8 (32.0)	46 (27.7)	0.516	0.472
Postoperative morbidity (%) (n = 166)	5 (29.4)	26 (38.8)	25 (43.9)	12 (48.0)	68 (40.5)	**2.550**	**0.026**^**†**^
Preoperative KPS score	75.3 ± 13.4	72.6 ± 13.2	71.8 ± 13.5	75.1 ± 13.3	73.7 ± 13.6	1.584	0.213
Preoperative excellent QOL (%)	7 (41.2)	29 (42.6)	25 (43.1)	11 (44.0)	72 (42.9)	0.143	0.893
Postoperative KPS score	62.2 ± 15.1	58.9 ± 16.3	59.5 ± 15.7	53.4 ± 16.4	58.5 ± 15.8	**6.274**	**0.038**^**†**^
Postoperative excellent QOL (%)	3 (17.6)	10 (14.9)	8 (13.8)	3 (12.0)	24 (14.5)	2.156	0.617
Follow-up KPS score	82.5 ± 9.1	80.9 ± 11.3	80.2 ± 11.8	79.8 ± 10.7	80.9 ± 10.6	0.708	0.533
Follow-up excellent QOL (%)	11 (68.8)	42 (64.6)	35 (63.6)	15 (65.2)	103 (64.7)	0.834	0.329
Follow-up patients No. (%)	16 (94.1)	65 (95.6)	55 (94.8)	23 (92.0)	159 (95.8)	1.356	0.237
Follow-up alive patients No. (%)	14 (82.4)	60 (89.6)	52 (91.2)	22 (88.0)	148 (89.2)	2.713	0.184
Follow-up median duration, mos	89.2	84.6	87.8	83.7	86.5	3.187	0.435
Follow-up morbidity (%) (n = 148)	4 (28.6)	15 (25.0)	14 (26.9)	6 (27.3)	39 (26.4)	1.154	0.159
Recurrence/Progression (%) (n = 148)	3 (21.4)	15 (25.0)	14 (26.9)	9 (40.9)	41 (27.7)	**5.804**	**0.015**^**†**^

^*^Values are presented as the number of patients (%) unless indicated otherwise. Mean values are presented as the mean ± SD. **NVS: Neurovascular Structure.

^**†**^p < 0.05.

### Surgical approach choice and outcomes

**Figure 10:**
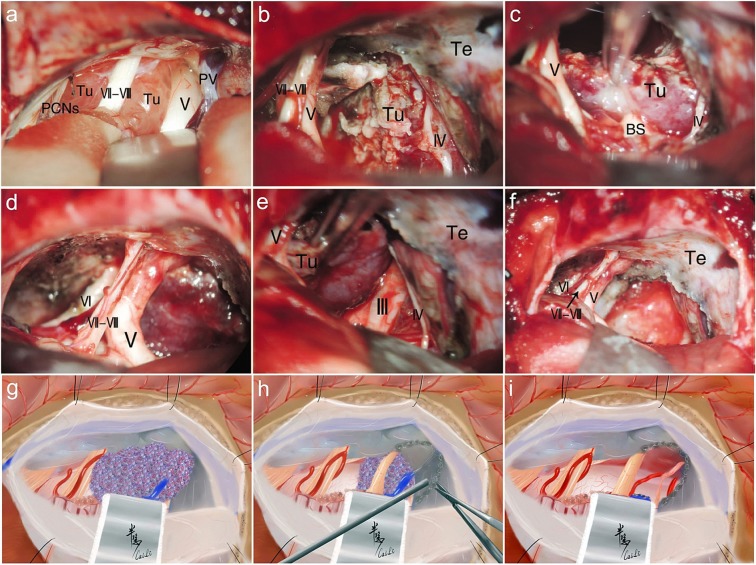
Intraoperative figures demonstrating steps of petroclival type resection via the RTTA. (**a**) Exposure of the tumor part of the superior and medial clival region after cerebellar retraction via the retrosigmoid approach. (**b**) Coagulation of the origin of tumor, performed intratumoral decompression, and then incision of the tentorium after removing the tumor located in the posterior fossa. (**c**) Separation of the adhesion between the tumor and brain stem. (**d**) Resection of the supratentorial part by piecemeal resection through incised tentorium cerebelli hiatus. (**e**) Separation of the adhesion between the suprotentorial tumor and CN III. (**f**) Gross total tumor resection and keeping well the neurovascular structures integrity. (**g–i**) The corresponding illustrations of main steps of RTTA. Tu, Tumor; PCNs, Posterior Cranial Nerves; PV, Petrosal Vein; Te, Tentorium; BS, Brainstem.

**Figure 11:**
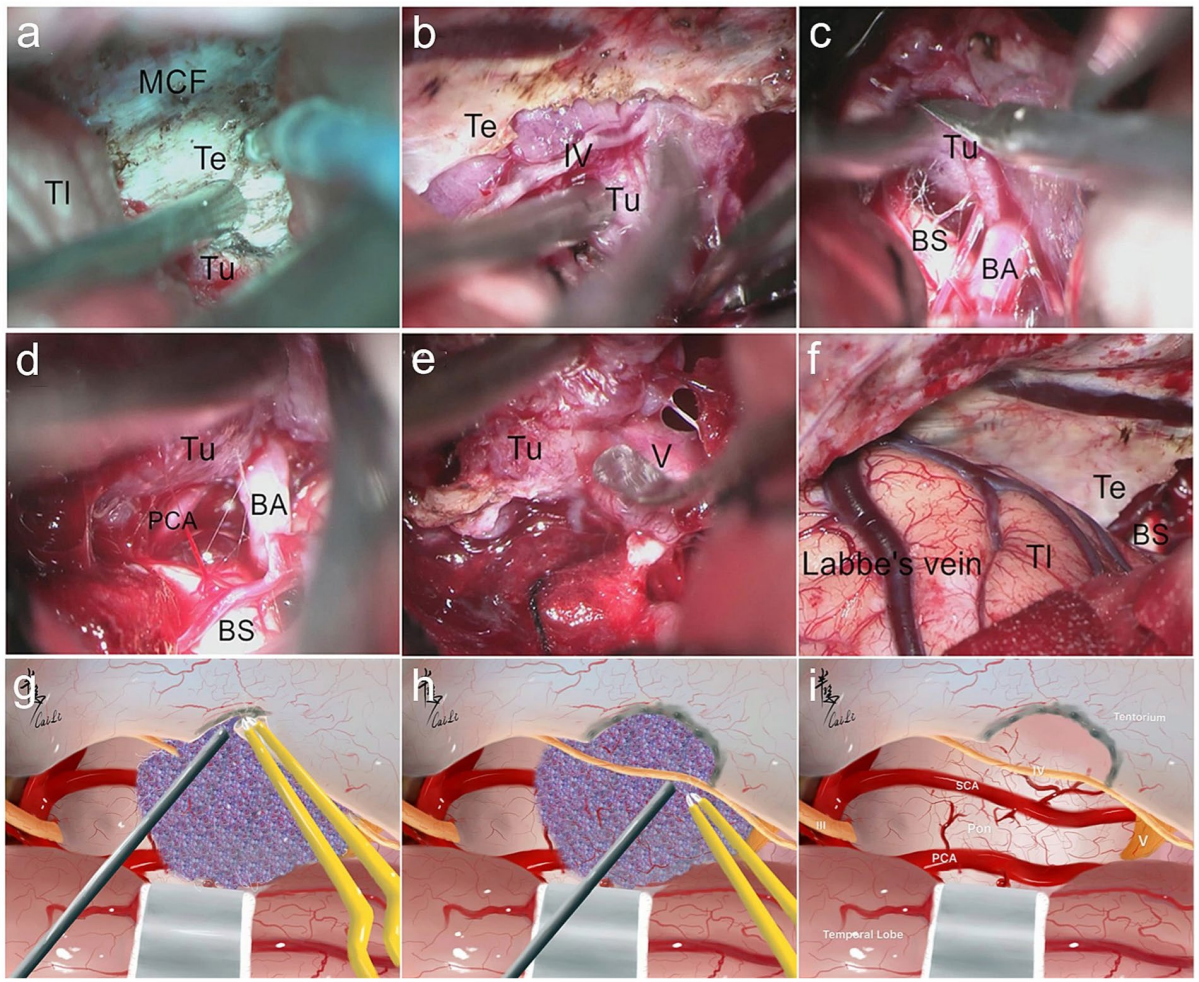
Intraoperative figures demonstrating steps of petroclivosphenoidal type resection via the STTA. (**a**) Exposure of the tumor of the supratentorial part, the middle fossa and tentorium after temporal lobe retraction. (**b**) Farther incision of tentorium and protection of CN IV during the tumor resection. (**c**) Sharp dissection of the arachnoid between the tumor and brain stem to protect the brain stem and basilar artery. (**d**) Farther dissection of the arachnoid between the tumor and brain stem. (**e**) Sharp dissection of the adhesion between the tumor and CN V. (**f**) Intact protection of Labbe vein and temporal lobe. (**g–i**) The corresponding illustrations of main steps of STTA. MCF, Middle Cranial fossa; Tl, Temporal lobe; PCA, Posterior Cerebral Artery; BA, Basilar Artery.

**Figure 12:**
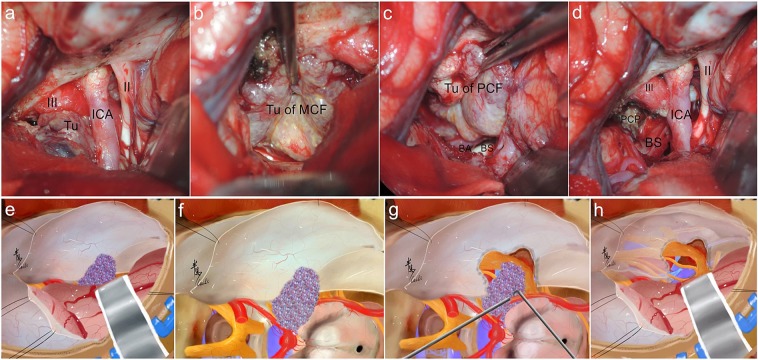
Intraoperative figures demonstrating steps of sphenopetroclival subtype II resection via the EPTA. (**a**) Exposure of the second interval and the third interval after temporal lobe retraction via regular pterional approach. (**b**) Tumor resection of the part of middle fossa. (**c**) Tumor resection of the part of posterior fossa. (**d**) Total tumor resection to protect the CN III and keeping well the brain stem and neurovascular structures integrity. (**e–h**) The corresponding illustrations of main steps of the EPTA. PCF, Posterior Cranial Fossa; PCP, Posterior Clinoid Process; Tu, Tumor; BS, Brainstem; BA, Basilar Artery.

Individualized surgical approaches were chosen based on the classification of tumor types (Table 3 and Fig. 9c). The overall GTR was achieved in 119 cases (70.8%), STR in 37 cases (22.0%), and PR in 12 cases (7.2%) (Fig. 9d). Concretely, for all of the CV type and 98.5% of the PC type, the RSA (including basic retrosigmoid approach, retrosigmoid transtentorial approach (Fig. 10) and retrosigmoid intradural suprameatal approach; BRSA/RTTA/RISA) was used with the GTR rate of 76.5% and 75.0%, respectively; for the PC-S type, the RSA was still mostly adopted (69.0%), followed by the subtemporal transtentorial transpetrosal approach (STTA) (Fig. 11) and presigmoid combined supra-infratentorial approach (PCA) (Fig. 6), with the GTR rate of 67.2%; for the S-PC I type and II type, the pretemporal trancavernous anterior transpetrosal approach (PTCA, 85.7%) (Fig. 7) and extended pterional transtentorial approach (EPTA, 81.8%) (Fig. 12) was mainly chosen, respectively, obtained the lowest GTR of 64.0% (P = 0.001) (Tables 2 and 3).

**Table 3:** Different surgical approach choice based on tumor type and tumor removal extent (n = 168).

Tumor type		n (%)	GTR (%)	Surgical approach* (%)						
				RSA			PCA	EPTA	PTCA	STTA
				BRSA	RTTA	RISA				
Clivus (CV)		17 (10.1)	13 (76.5)	17 (100)	—	—	—	—	—	
Petroclival (PC)		68 (40.5)	51 (75.0)	53 (77.9)	14 (20.6)	—	1 (1.5)	—	—	
Petroclivosphenoidal (PC-S)		58 (34.5)	39 (67.2)	—	24 (41.4)	16 (27.6)	6 (10.3)	—	—	12 (20.7)
Sphenopetroclival (S-PC)	S-PC I	14 (8.4)	16 (64.0)	—	—	2 (14.3)	—	—	12 (85.7)	
	S-PC II	11 (6.5)		—	—	2 (18.2)	—	9 (81.8)	—	—
**Total**		**168**	**119 (70.8)**	**128**			**7**	**9**	**12**	**12**

^*^RAS retrosigmoid approach, **BRSA** basic retrosigmoid approach, **RTTA** retrosigmoid trantentorial approach, **RISA** retrosigmoid intradural suprameatal approach, **PCA** presigmoid combined supra-infratentorial approach, **EPTA** extended pterional transtentorial approach, **PTCA** pretemporal trancavernous anterior transpetrosal approach, **STTA** subtemporal transtentorial transpetrosal approach.

There were no intraoperative death cases; two GTR patients (1.2%) died within 48 hours after operation due to pulmonary infection and cerebral infarction, respectively. The mean duration of the surgeries was 6.4 ± 1.8 hours (range 4.2–9.0 hours). The mean and median postoperative length of stay (LOS) was 15.1 ± 5.6 and 16.0 days (range 6–42 days), respectively. The main complications (46 cases, 27.7%) included hypostatic pneumonia, CSF leakage, intracranial hematoma, cerebral infarction, intracranial infection, aggravated brain edema, hydrocephalus, epilepsy, and alalia (Fig. 9b). The most common morbidities (68 cases, 40.5%) were new or deteriorated neurological disorders. Meanwhile, the immediate recovery or relief of previous neurological disorders (62 cases, 37.3%) was also remarkable (Table 1). However, the postoperative KPS score had significantly decreased to 58.5 ± 15.8 (range 20–90), mainly due to surgical morbidities and complications. Compared with other types, the patients of S-PC type suffered a longer surgical duration time (P = 0.006) and LOS (P = 0.017), a lower KPS score (P = 0.038) and a higher morbidity rate (P = 0.026), without a significant difference in complications (P = 0.472) or excellent QOL rate (P = 0.893), postoperatively (Table 2). The GKS was recommended routinely for nGTR patients and usually performed 1–3 months postoperatively for residuals, depending on the patients’ recovery.

### Long-term follow-up and prognosis

**Figure 13:**
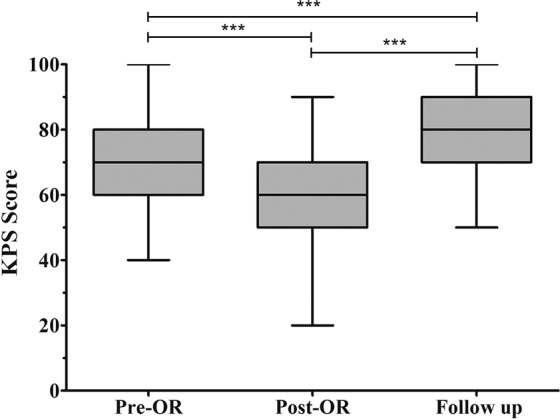
Box plot showing the preoperative, postoperative, and follow-up KPS score. ***p < 0.001.

**Figure 14:**
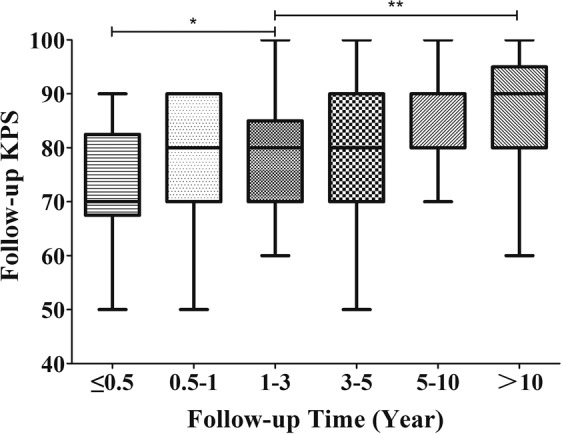
Box plot showing different period groups of KPS score during the follow up. *p < 0.05, **p < 0.01.

**Figure 15:**
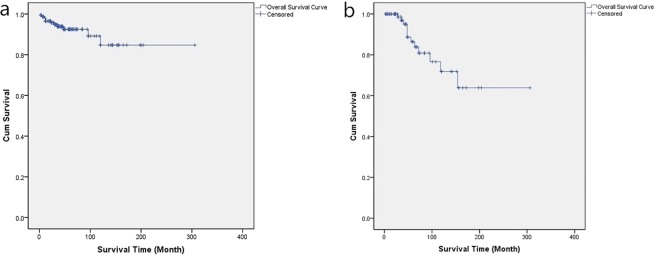
Kaplan-Meier survival analysis illustrating the survival time. (**a**) The Overall survival (OS) time of the PCM patients in the latest follow-up. (**b**) The R/P-free survival (R/P-FS) time of the alive PCM patients in the latest follow-up.

In the latest follow-up evaluation, 159 cases of the 166 discharge patients (95.8%) had recorded with the median period of 86.5 months (range 12–246 months) including over 3 years in 97 cases (61.0%), over 5 years in 54 cases (34.0%), and over 10 years in 20 cases (12.6%). During the follow-up, 11 patients (6.9%) died including 5 cases of progress, 4 cases of recurrence and 2 cases of irrelative diseases. In the remaining cases, the symptoms had improved or recovered in 84 cases (56.8%), and the manifestations of 39 cases (26.4%) only had not changed obviously (Table 1) which had significantly decreased compared with the postoperative morbidity (t = 7.813, P < 0.001). The latest KPS score showed an average of 80.9 ± 10.6 (range 50–100), which was higher than both of the preoperative (t = 5.322, P < 0.001) and postoperative (t = 13.220, P < 0.001) KPS score (Fig. 13). The latest excellent QOL rate was 64.6%, significantly higher than that of the preoperation (42.9%) and postoperation (14.5%) (χ^2^ = 76.768, P < 0.001). However, there was no difference between the tumor types in follow-up morbidity rate, KPS score and excellent QOL rate (Table 2). Additionally, to compare the variation of the KPS score in different follow-up periods, these cases were divided into six groups, depending on follow-up duration including Group A (within 6 months) with a KPS of 72.0 ± 13.9, Group B (6–12 months) with a KPS of 79.1 ± 14.2, Group C (1–3 years) with a KPS of 78.7 ± 11.6, Group D (3–5 years) with a KPS of 80.8 ± 11.9, Group E (5–10 years) with a KPS of 83.6 ± 11.7, and Group F (over 10 years) with a KPS of 85.3 ± 13.1. The comparison shows that the differences in KPS score between Group A and Group C (t = 1.847, P = 0.041) and between Group C and Group F (t = 2.799, P = 0.007) were statistically significant (Fig. 14). Excepting two unrelated death cases, the overall survival (OS) of PCM patients showed that the mean follow-up survival time was 269.5 ± 12.3 months, with an OS rate of 96.5% at 1 year, 93.8% at 3 years, 89.2% at 5 years, and 84.7% at 10 years (Fig. 15a).

The overall R/P rate was 25.7% with 41 cases in the latest follow-up, with a rate of 40.9% in S-PC type, significantly higher than other types (P = 0.015) (Table 2). In the GTR group, 112 cases of 117 discharged patients were followed up on with the median time of 82.0 months (range 12–246 months). And 20 cases of recurrence (17.9%) were found with the median time of 30.7 months postoperatively. In non-GTR (nGTR) group, 47 cases of 49 discharged patients were observed including 12 cases of conservative observation and 35 cases of subsequent therapy, with the median follow-up time of 62.8 months (range 12–200 months). And 21 cases of progress (44.7%) were found with the median time of 27.6 months postoperatively. While the progress rate of the conservative observation group (7 cases, 58.3%) was significantly higher than that of the subsequent therapy group (14 cases, 40.0%) (χ^2^ = 4.524, P = 0.037) in nGTR cases. The mean R/P-free survival (R/P-FS) time was 227.3 ± 20.3 months, with an R/P-FS rate of 98.5% at 1 year, 95.0% at 3 years, 88.7% at 5 years, and 63.8% at 10 years (Fig. 15b).

Furthermore, the differences between the GTR group and the nGTR group were compared (Table 4). The mean values of surgical duration, postoperative LOS, postoperative complications, postoperative morbidity, postoperative KPS score, and postoperative excellent QOL were not significantly different. But the follow-up KPS score (P = 0.043), excellent QOL (P = 0.026), R/P rate (P = 0.001) and R/P-FS mean time (P = 0.032) did show statistical significance.

**Table 4:** Surgical and follow-up outcomes compared based on the EOR.

Variable		GTR (n = 117)	nGTR (n = 49)	Total (n = 166)	*t/χ*^2^	p Value
Surgical duration, hrs		6.6 ± 1.6	6.2 ± 2.2	6.4 ± 1.8	0.167	0.233
Mean of postop LOS, dys		13.3 ± 9.0	17.7 ± 8.9	15.1 ± 8.6	2.554	0.056
Postop-complication	Yes	33	13	46	0.138	0.417
	No	84	36	120		
Postop-morbidity	Yes	49	19	68	0.562	0.279
	No	68	30	98		
Postop QOL	KPS score	57.3 ± 15.5	59.7 ± 16.6	58.5 ± 15.8	1.596	0.091
	Excellent	19	5	24	0.079	0.482
	Non-excellent	98	44	142		
Follow-up QOL (n = 159)	KPS score	83.6 ± 8.7	78.2 ± 14.6	80.9 ± 10.6	**3.371**	**0.043**^**†**^
	Excellent	74	29	103	**4.509**	**0.026**^**†**^
	Non-excellent	38	18	56		
Recurrence/ Progress (n = 159)	Rate (%)	14.1	44.7	25.7	**12.432**	**0.001**^**†**^
	FS time, mos	232.7 ± 18.6	198.5 ± 10.4	227.3 ± 20.3	**2.136**	**0.032**^**†**^

^**†**^p < 0.05.

## Discussion

PCMs account for 3–10% of PCF meningiomas, which comprise about 0.15% of all intracranial tumors^8^. The growth patterns of lesions were unpredictable and multifarious, leading to serious neurofunctional deficits and unfavorable prognoses. In the cases observed in this study, the median symptom duration was 41.2 months and the mean lesion size was 44.0 mm, with most lesions (96.4%) classified as large or giant. The ideal treatment strategy of PCMs continues to be a matter of controversy due to the low incidence, variable biological behavior, incredible size and anatomical involvement of critical neurovascular structures causing radical removal risky^9^. Recent literature has revealed a tendency of choice of less aggressive approaches^10–16^, whereas the application of complicated skull base approaches was more restricted and specific^8^. Adhering to this concept, most approaches we chose were simple and practical, less injurious, and faster recoverable in order to explore an appropriate treatment strategy based on an accurate tumor classification to find the balance point of the increase of EOR and preserve of neurological functions.

### Classification of PCMs and the choice of surgical approach

The correlation between tumor classification and surgical approach choice has been recognized gradually provide an efficient guidance to individualized therapy^2^. Therefore, the precise classification is the starting point for the choice of a tailored surgical strategy. Multiple classifications for tumors in the petroclival region have been described^1–3,5,6^ of which the value and guidance were undeniable. In our study, the choice of the ideal treatment which enriched and replenished the tumor classification was founded on the deeper evaluation of the outcomes and better realization of the role of each approach we adopted. To be more specific, the unique aspects of our study are the following: (1) The classification is established on the changes of the pathologic anatomy, based not only on the radiological features but also on the intraoperative identification and the surgeon clinical experience; (2) The PC-S type, defined for the first time, is emphasized to be different from the S-PC type based on the origin of dural attachment and the growth pattern; (3) The S-PC type is classified into two subtypes initially, mainly based on the relationship between the lesion and the CS invasion; (4) The different types are associated with different clinical characteristics and outcomes, helping to semiquantitatively anticipate the prognosis.

The BRSA/RTTA was the preferred approach for the CV type and PC types (Figs. 2 and 3) and achieved a high GTR rate (76.5% and 75.0%, respectively). Compared with the transpetrous approaches, the BRSA offers an easy and fast craniotomy to provide a satisfactory view of the clivus, petrous apex and tentorial incisure without excessive cerebellum traction^17^, avoiding petrous bone drilling and venous sinus handling. Meanwhile, this approach offered a wider surgical corridor to prior and convenient exposure of the CNs–tumor–brainstem interface and the dural attachment without drilling of internal structures of petrous bone^11^. However, most of the large and giant lesions in our study invaded the tentorium or even ruptured into the supratentorial compartment. The RTTA, which was combined with the transtentorial approach based on the BRSA (Fig. 10), improved the extent of exposure to the petroclival region by adding operative intervals to resect supratentorial lesions^18^. The RTTA was considered as an excellent approach to reach the ventrolateral brainstem and the petroclival region for providing a better superoventral exposure of the ventrolateral brainstem than BRSA^19^ verified our opinion and choice. In our experience, the tentorium would be incised regularly to explore the upper surface of the tentorium, if the lesion is attached to the tentorium, even without rupturing into the supratentorial space (as judged from MRI), because some lesions has grown on the tentorial surface. Consequently, the BRSA/RTTA is the optimal choice for the CV type and PC type especially in the following situations: (1) The tumors mainly locate in the PCF; (2) The tumors have invaded into the whole clivus and extended from the foramen magnum to the dorsum sellae; (3) The tumors originate from the tentorial edge, attached to the PF and petrous apex, and extended toward the supratentorium.

As for the PC-S type, the strategy included a posterior approach (69.0%, RTTA/RISA) or an anterolateral approach (31.0%, STTA/PCA), depending on the origin of the dural attachment and the main lesion location. (1) If the dural attachment derived from the middle-upper clivus and mainly located in the PCF, or extended through the tentorial incisura straddling the supra-infratentorium, the RTTA was still the optimal choice. (2) If the tumor invaded into the MCF through the MC (Fig. 4), the RISA was selected and the tumors without CS invasion could be treated with GTR. In our opinion, an incision of the tentorium or drilling the IAM margin could add a new operative corridor and working angle from the PCF to direct access to the dural attachment site on the dorsal petrous bone providing an easy opportunity to disrupt the tumor blood supply.

If the other cases of the PC-S type showed the following pathological anatomy characteristics, the employment of the STTA was suggested: (1) The main origin of the dural attachment typically locates at the upper clival region and the lesions straddle at the petrosal apex, with the main body located in the MCF or extended into the supratentorial compartment; (2) The portion located in the PFC is medial to CN VII and not lower than the IAM; (3) The lesions have adhered to or even invaded the posterior wall of the CS; (4) The lesion invaded into the MC with the main dysfunction of trigeminal nerve (Figs. 5 and 11). With the subdural intermittent and the temporal lobe traction, the interface of the lesion and brainstem could be observed directly from the anterior-petrosal view and the clival dural attachment could be observed from a posterior-petrosal angle, providing multiple perspectives removal. Then the tentorium is incised to acquire a better exposure of the subtentorial structures. Restricted by the temporal lobe and petrous ridge, the petrous apex could be drilled to increase the exposure range to resect lesions adjacent to petrous bone and the middle clivus if needed acquiring an ideal exposure of the dural attachment from the supratentorial to infratentorial direction and from the petrosal dorsum to the middle-lower clivus. The PCA is considered the preferred method in the following cases (Fig. 6): (1) The lesions are too large to grow sprawlingly and involve of the skull base structures extensively; (2) The lesions often extend inferior to the IAM and lateral to the petrosal bone; (3) The tentorium has been invaded widely. However, considering the disadvantages (e.g. the relatively complicated procedure, long-time consuming, and high risk of complication), this complex approach has been used more restrictedly and was replaced with the combination of STTA and RSA gradually in our study. The GTR rate of this type was down to 67.2% with a higher comorbidity due to brainstem edema than the other types (Table 2) which mainly influenced the EOR. Thus, the tumor rim or capsula on the brainstem may be retained to avoid clinical deterioration due to postoperative edema aggravation or vasospasms.

As for S-PC subtype I, the PTCA was mainly applied (Fig. 7) to create an adequate MFC-to-PCF, medial-to-lateral, and superior-to-inferior route for resection, combining with the pretemporal transcavernous approach (PTA) and the anterior transpetrosal approach (ATA). The surgical route could reach the suprasellar, interpeduncular, and upper-third clival regions through the PTA. The ATA provides a corridor to the PCF to remove the portion located medial to the IAM by drilling the anterior petrous apex. In conclusion, PTCA revealed the following advantages for subtype I: (1) The meningio-orbital artery is used as the starting point to access the interstice between the dura mater and the lateral wall of CS; (2) The epidural traction of temporal lobe could maintain the draining veins to reduce postoperative edema; (3) The individualized exposure of the CS and abutting structures could be performed to meet sufficient lesion exposure, maximal EOR, and less surgical injury. As for tumors of S-PC subtype II, the EPTA was mainly adopted (Figs. 8 and 12) which is based on the expanded pterional approach and combining with the transtentorial approach if necessary. With sufficient separation and traction of temporal lobe, the EPTA could achieve the exposure of the sphenoid ridge and anterior clinoid process anteriorly, the trigeminal initial part in the brainstem posteriorly, the trigeminal ganglion laterally, and the posterior clinoid process medially. Therefore, the EPTA was suitable for subtype II especially in the following conditions: (1) The lesion exceeded the dorsum sellae, infiltrated the tentorium, and extended into the supratentorial space; (2) The main portion locates in the MCF, invades into the CS, and impaires the sinus wall; (3) The portion located in the PCF is not lower than the middle clivus. Unsatisfactorily, the overall GTR rate was sharply down to 64.0% due to the critical neurovascular structures of the CS, through which the ICA and multiple CNs run^20^. Thus, the portion of the lesion within the CS that is not wrapped with CNs/the ICA and that has a soft consistency could be removed maximally. But the enclosed parts or those with a hard consistency within the CS had to be left aside to avoid serious complications.

### Surgical outcomes

The overall GTR rates of PCMs reported in the literature were ranging from 20% to 75%^6,10–12,21–26^, and in this study, we obtained the GTR rate of 70.8%. Although STR with radiosurgery to avoid operative morbidity was recommended^22^, Al-mefty *et al*. pointed out that complete resection was still the primary choice for the radical treatment of most PCMs that could mitigate recurrence, achieve low morbidity in major cases, and improve the functional outcome^9,16,27^. Meanwhile, Samii *et al*. indicated similar points the EOR was the most important predictor of outcome, and every effort should be made to remove the tumor completely at initial surgery^16^. Adhering to this concept and applying our experience of over 20 years, we explored the balance point between minimum morbidity and maximum tumor removal to improve the QOL.

In our study, the comparison based on the EOR (Table 4) showed that there was no difference between the GTR group and the nGTR group in surgical duration and postoperative LOS, implicating radical resection did not increase operation time or prolong the postoperative LOS. No correlation was found between the EOR and the complication rate, morbidity, postoperative KPS, or excellent QOL, which means the postoperative complication rate and morbidity were not higher and postoperative neurological functions were not worse in the GTR group than in the nGTR group. More importantly, the recent neurological status of the GTR group was better than in the nGTR group in terms of the follow-up KPS (P = 0.043) and excellent QOL (P = 0.026). Meanwhile, the R/P rate of GTR group was lower and mean R/P-FS time was longer than that of the nGTR group, respectively. All of these outcomes demonstrated that GTR significantly decreased the R/P rate, prolonged the R/P-FS time and improved the recent neurological status which indicated that the radical removal was feasible, necessary, and the optimal surgical management for PCMs. Therefore, we advocate that one should try to achieve radical lesion removal in the initial operation, as long as the neurological status and QOL of the patient are ensured.

The mean surgical duration and postoperative LOS was 6.4 ± 1.8 hours and 15.1 ± 8.6 days respectively, reflecting a lesser time-consuming operation and faster recovery. The S-PC type was associated with longer surgical times and LOS, reflecting much tougher surgeries and more severe postoperative reactions than other types. In the literature, the incidence of CN deficits varies between about 40% and 70%^9,20,27–29^. Despite the relatively high rate of postoperative morbidity (40.5%), the follow-up morbidity significantly decreased to 26.4% (P < 0.001) meaning most of postoperative neurological deficits were temporary and transient in our study. A major improvement in CN deficits was observed, especially in CN V–XII (Table 1). Therefore, the neurological status improved gradually or even recovered during the follow-up, even when radical resection had been applied, which confirms the feasibility of radical resection. For the S-PC type, the postoperative morbidity was higher (P = 0.026) and KPS score was worse (P = 0.039) than for other types respectively because of more involvement of the MC, CS, and CNs in this type, but the morbidity during the follow-up was no difference between the types, confirming the recovery of the S-PC type was not worse than for others. The complication rate was 27.7%, due to the lesions frequent adherence to critical neurovascular tissue which was associated with greater surgical complication rates. Fortunately, statistically significant improvements were observed compared the follow-up KPS score with the postoperative KPS score (P < 0.001). There was no difference of complication between the types, meaning the different types and surgical approaches did not increase the complication incidence. However, how to improve the surgical techniques, protect the intactness of neurovascular structures, and reduce the complication is still a challenging issue for neurosurgeons.

### Follow-up and prognosis

A systematic follow-up was continued with a high follow-up rate (95.8%) and long period (the median period of 86.5 months with the longest period of 246 months). Both of the latest KPS score and excellent QOL rates had significantly increased respectively (P < 0.001), showing the neurological status and QOL improved notably (Fig. 13). With subsequent follow-ups, the KPS score increased gradually in different groups, which meant the QOL recovered gradually as a whole (Fig. 14). The KPS score of Group C was higher than that of Group A (P = 0.041), proving that the QOL was obviously improved over one year postoperatively. Meanwhile, within 10 years, the KPS scores were almost stable and the QOL did no longer show a prominent improvement, because there is no difference between Groups C, D, and E. However, the KPS score of Group F was significantly higher than that of Group C (P = 0.007), which suggests that if the patient lived over 10 years postoperatively, the QOL would be better than ever before. There was no difference between the different types in their follow-up KPS scores and excellent QOL rates, showing that the patients recovered without intergroup differences, although the postoperative KPS score in the S-PC group was much lower (Table 2).

In our follow-up, the R/P rate of 25.7% was obtained with 17.9% of recurrence in GTR and 44.7% of progress in nGTR and the S-PC type obtained higher R/P rate than others (P = 0.015) due to the low GTR rate, which verified that the higher R/P rate was significantly associated with a lower EOR^9,21,22,30^. In our study, the long-term R/P-FS rate was 98.5% at 1 year, 95.0% at 3 years, 88.7% at 5 years, and 63.8% at 10 years, with an average of 227.3 months certified the benefit of radical resection. The progress rate of the subsequent therapy group was significantly lower than that of the conservative observation group (P = 0.037) in nGTR cases, obviously indicating that tumor progress could be effective controlled by subsequent therapy (GKS/reoperation). As Al-mefty *et al*.^9^ suggested, complete resection mitigated the recurrence in a majority of the cases, with low mortality and morbidity and an improvement in the functional outcome. Therefore, Although, not all PCMs could be completely removed, GTR should be attempted in most cases or at least the maximum tumor resection in the initial surgical treatment. Adjuvant therapies are only used as subsequent treatment for tumor residuals or aggressive pathology.

### Proposed treatment strategy for PCMs

**Figure 16:**
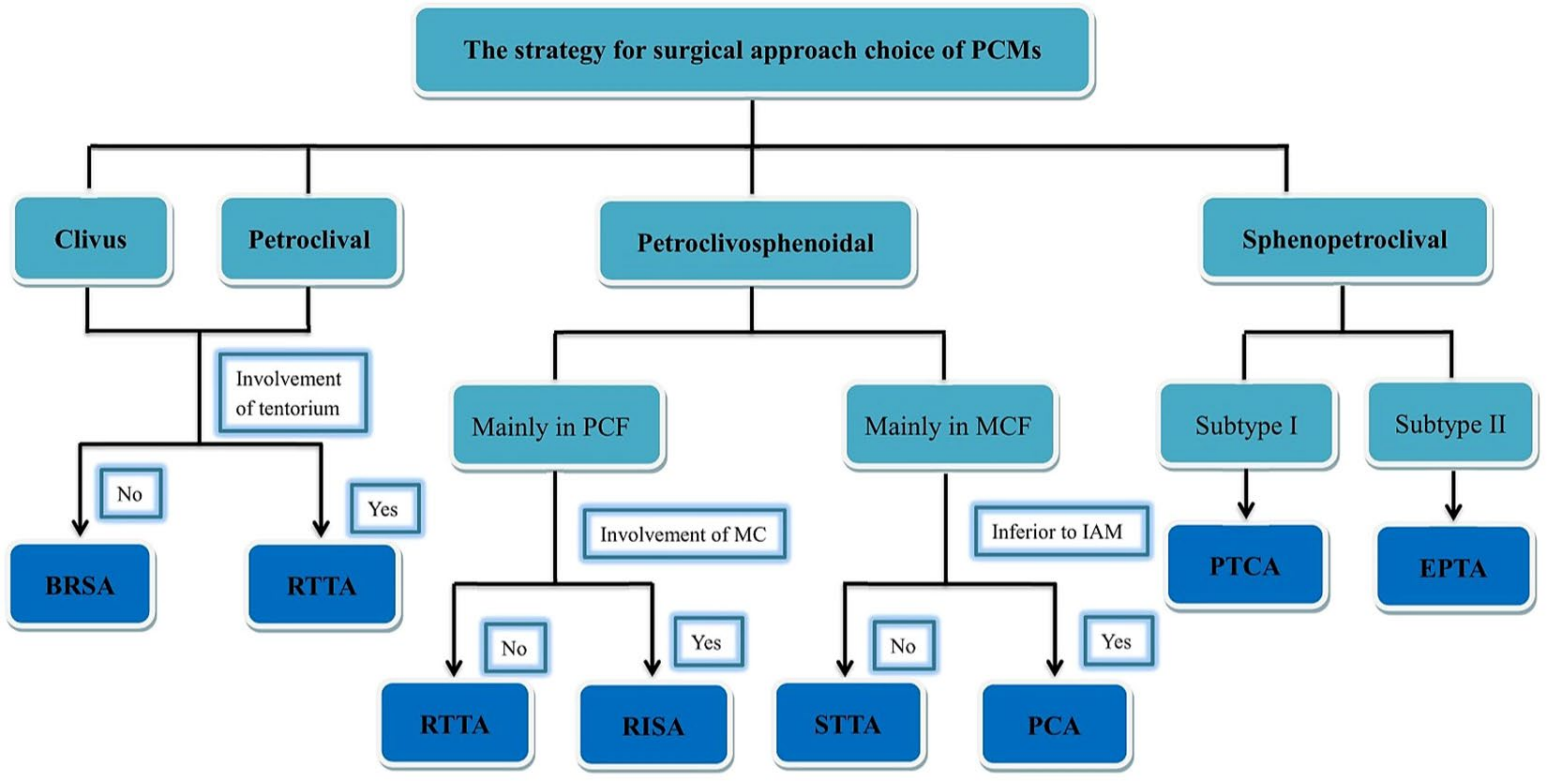
Structure chart illustrating the strategy for individualized surgical approach choice based on the different tumor type.

Surgery is still the primary treatment for PCMs, be it with the necessary subsequent therapies. It is paramount to choose an appropriate surgical approach and improve the GTR rate by the classification of the tumor type based on the pathological anatomic variation. Despite the high incidence of early postoperative complications, during the continuous follow-up period a significant neurological and QOL improvement was observed. The individualized choice of approach for different types is proposed in Fig. 16. The strategy for each type is considerably diverse due to the different patterns, especially for the PC-S type and the S-PC type. The treatment of the CV type and PC type are ideal with a simple approach choice. But for the PC-S type and S-PC type, individualized approaches are needed, considering the priority of the QOL and the complexity of the dural attachment and involved structures. The proposed classification of PCMs could guide the choice of the most suitable approach to deal with PCMs in order to maximize the tumor removal. Our classification, matching the change in pathologic anatomy in the petroclival region, is proposed for the first time and displays a close correlation with individualized approaches, which could obviously increase the GTR rate, decrease the complications and morbidity, and even improve the long-term QOL.

### Limitations of study

The present study was a retrospective review of PCMs which are benign and relatively rare lesions leading to a potential selection biases because of the nonrandomized retrospective study design and relatively small cohort size. Meanwhile, we obtained a relative long-term follow-up period, but part of patients were lost to follow-up with the prolongation of the follow-up time. However, we have only focused on the PCMs in the present study, and the results offered considerable value toward understanding the clinical characteristics of PCMs and guiding neurosurgeons in choosing the optimal treatment strategy for patients with PCMs.

## Conclusions

Favorable outcomes and acceptable morbidity were achieved with the microsurgical management of PCMs. It is rational to recommend that GTR should be achieved during the first treatment of PCMs, if it can be accomplished with minimal morbidity. The choice of the specific approaches serving the goal of a safe, uncomplicated, and less aggressive access to the petroclival region based on the tumor classification improved the GTR and QOL for the patients. Meanwhile, the differences between the tumor types in diverse clinical characteristics, the EOR and R/P were verified. Sufficient individualized assessment and suitable approach choice should be based on the tumor classification in order to increase the therapeutic efficacy, decrease the morbidity, and improve the prognosis for the patients.
